# Antidepressant-Like Effects of Erythropoietin: A Focus on Behavioural and Hippocampal Processes

**DOI:** 10.1371/journal.pone.0072813

**Published:** 2013-09-03

**Authors:** Meagan Osborn, Nazneen Rustom, Melanie Clarke, Darcy Litteljohn, Chris Rudyk, Hymie Anisman, Shawn Hayley

**Affiliations:** Department of Neuroscience, Carleton University, Ottawa, Ontario, Canada; Radboud University, Netherlands

## Abstract

Depression is a chronic and debilitating condition with a significant degree of relapse and treatment resistance that could stem, at least in part, from disturbances of neuroplasticity. This has led to an increased focus on treatment strategies that target brain derived neurotrophic factor (BDNF), synaptic plasticity and adult neurogenesis. In the current study we aimed to assess whether erythropoietin (EPO) would have antidepressant-like effects given its already established pro-trophic actions. In particular, we assessed whether EPO would diminish the deleterious effects of a social stressor in mice. Indeed, EPO induced anxiolytic and antidepressant-like responses in a forced swim test, open field, elevated-plus maze, and a novelty test, and appeared to blunt some of the negative behavioural effects of a social stressor. Furthermore, EPO promoted adult hippocampal neurogenesis, an important feature of effective antidepressants. Finally, a separate study using the mTOR inhibitor rapamycin revealed that antagonizing this pathway prevented the impact of EPO upon forced swim performance. These data are consistent with previous findings showing that the mTOR pathway and its neurogenic and synaptogenic effects might mediate the behavioral consequences of antidepressant agents. Our findings further highlight EPO as a possible adjunct treatment for affective disorders, as well as other stressor associated disorders of impaired neuroplasticity.

## Introduction

Affective disorders are associated with structural brain changes, such as reduced hippocampal volume [1], which might stem from stressor-provoked reductions of neurogenesis [2–4]. In this regard, particular attention has been afforded the involvement of diminished brain-derived neurotrophic factor (BDNF) in depression as this growth factor ordinarily enhances neurogenesis and promotes neuronal cell survival and neurite growth [5,6]. Studies in animals have shown that stressors reduce hippocampal BDNF, and its administration attenuated the depressive-like behavioural effects elicited by stressful events [4,6–9]. In humans, serum BDNF levels have frequently been found to be reduced in depressed individuals, but elevated following successful pharmacotherapy [10,11]; however, contradictory reports exist in this regard [12]. In addition to BDNF, other trophic factors, including glial derived neurotrophic factor (GDNF) and fibroblast growth factor (FGF-2), might also play a role in depressive disorders [13,14].

These findings suggest that it would be beneficial to administer trophic factors such as BDNF in the treatment of depression. However, BDNF may have untoward side effects, including moderation of pain pathways [15–17]. Moreover, BDNF and other neurotrophic proteins do not appreciably cross the blood brain barrier (BBB), making the usefulness of peripheral administration questionable [18,19]. Nonetheless, in rodents peripheral BDNF treatment increased hippocampal neurogenesis and BDNF protein levels, and reduced signs of anxiety in several behavioural tests [20].

Erythropoietin (EPO) is a hematopoietic growth factor that readily crosses the BBB and is routinely used clinically to treat anemia [21]. This trophic cytokine has not been assessed as extensively as other growth factors in the context of depression, but recent studies have implicated EPO as having clinical potential. Pre-clinical data suggested that EPO might be an agent to promote neuronal recovery, having neuroprotective consequences in models of stroke and traumatic brain injury [21]. Likewise, EPO induced cognitive improvements in healthy, as well as neuronally compromised animals [21,22]. The available data are admittedly sparse, but there have been reports indicating that EPO may have antidepressant and potentially even anxiolytic actions [23]. In this regard, it was reported that EPO induced antidepressant-like effects in a forced swim test and altered novelty-induced hypophagia (NIH) [24]. Furthermore, EPO levels were elevated in the dentate gyrus (DG) of the hippocampus after electroconvulsive seizure treatment in rats [24]. Human brain imaging studies indicate that EPO directly improves hippocampal function [23] and modulates brain responses to emotional information in a manner similar to that of conventional antidepresssants in the absence of hematological changes [25,26].

It was reported that EPO and/or EPOR was present on neurons within the hypothalamus, hippocampus and neocortex of adult rodents [27]. As the non-hematopoietic, carbamylated form of EPO, c-EPO, also robustly influences CNS activity, it is likely that EPO influences brain functioning independent of any effects on red blood cells. In fact, hippocampal EPO levels were elevated after treatment with an antidepressant or electroconvulsive stimulation [24], and the administration of EPO induced BDNF expression and adult hippocampal neurogenesis [28,29].

In light of these findings EPO may promote antidepressant effects by inducing BDNF expression or by directly stimulating trophic pathways involving phosphatidylinositol-3-kinase (PI3-K), Akt/protein kinase-B, MAP kinases, and STAT5 [30]. However, EPO is also known to influence peripheral cellular growth through the mammalian target of rapamycin (mTOR) pathway [31]. Given the recent findings implicating the mTOR pathway in the rapid and prolonged anti-depressant actions of the NMDA antagonist, ketamine [32], we hypothesize that this pathway also contributes to the behavioural effects of EPO. Hence, the present study assessed the impact of EPO upon a range of depressive- and anxiety-like behaviours elicited by a social defeat stressor in mice, and whether EPO would augment hippocampal neurogenesis. As well, we assessed whether the mTOR inhibitor, rapamycin, would modify the behavioural effect of EPO in the forced swim test (FST).

## Methods

### Experimental Animals

Sixty-four male CD-1 mice (Charles River), 10-12 weeks of age, served as experimental subjects. In addition, 16 CD-1 retired breeders (Charles River) were used as aggressive conspecifics in the social defeat paradigm. A separate study involving 32 male CD-1 mice (Charles River) was conducted to assess the impact of the mTOR pathway inhibitor, rapamycin, on the behavioural effects of EPO in the FST. Animals were individually housed in the non-stressed condition in standard (27 × 21 × 14 cm) polypropylene cages. All animals were maintained on a 12-h light/dark cycle with lights on at 08:00 h. Mouse chow (Charles River diet, 5071) and water was provided *ad libitum*, and room temperature was maintained at approximately 21°C. All procedures were approved by the Carleton University Committee for Animal Care and were conducted in adherence to guidelines set out by the Canadian Council for the Use and Care of Animals in Research.

### Injection Treatments

In order to label dividing cells, on the first day of the experiment prior to any other treatment being administered all mice received a single intraperitoneal (i.p) injection of bromodeoxyuridine (BrdU, 200 mg/kg; Sigma-Aldrich, lot 060M1224V). Thereafter, mice received either i.p. injection of EPO (R&D systems; recombinant mouse EPO; lot # EUP0409111) or saline (Sigma-Aldrich; lot RNBB9031) 3 times a week for 2 weeks. EPO was delivered at a dose of 5000 U/kg (which was calculated to be ~40 µg/kg). Both the saline and the EPO were injected at a volume of 0.4 ml.

### Social Defeat Stressor

As depicted in Table S1, half of the EPO and half of the saline treated mice (n = 16/group) were subjected to the social defeat stressor from Day 1 to Day 14, whereas non-stressed mice remained undisturbed in their home cages. The stressed animals were placed in a novel cage with an aggressive CD-1 bully mouse, with a mesh divider keeping them separated. Once a day, at random times after the completion of any required behavioural tests, the divider was lifted and the mice were allowed to interact physically. Once a mouse showed submissive behaviours or excessive fighting occurred (continuous biting), the divider was put back into place. Submissive behaviours were defined as standing on back paws while waving front paws in the air, or continuously cowering in a corner and then squeaking and running when the dominant mouse came near. If the mouse was not clearly defeated by the end of 5 minutes the divider was put back and a new bully mouse was introduced the next day. All interactions were recorded, including latency to fight and submit, submission style, and the bullies’ behaviour (fighting style, aggressive tail wagging); any injuries were recorded and carefully monitored. No mice needed to be removed from the study due to injuries over the course of the 14-day stressor regimen.

### Behavioural Testing

Behavioural testing occurred on the 7th, 10th and 13th days of the experiment (i.e., during the second week of the study; see Table S1). As well, each test session was conducted early in the day just before the daily interaction with the bully mouse and included assessment in the FST, open field (OF) and elevated plus maze (EPM), as well as performance on a novelty task. Separate mouse cohorts from each group were tested on each of the aforementioned test days. As statistical analyses revealed no significant cohort/day differences, data were pooled over the three testing sessions. As well, tests were presented in a predetermined random order to preclude order effects. This sequence of spaced testing allowed assessment of performance on a series of depression-relevant tasks, without “overloading” mice and promoting stress effects related to the behavioural tests themselves. On the behavioural assessment days, testing commenced no later than 1 PM and terminated prior to the application of the daily social stressor.

### Forced Swim Test

Mice were individually placed for 10 minutes in a 2/3rd filled 2000 mL beaker with 25 ± 1°C water. The mice could not escape, and their feet could not touch the bottom. After each swim, mice were lightly towel dried and introduced back into their home-cage, which was then placed for 15 min on a heating pad set to medium heat. The water in the beaker was changed between each test. Each FST session was video recorded and later scored for time spent swimming or climbing (pedaling or circular movements around the beaker or active attempts to climb the beaker wall), and time immobile (lack of movement beyond those movements necessary to maintain balance).

### Open Field Test

Mice were individually placed in the corner of a 30 cm^3^ open field (OF), which was constructed from opaque Plexiglas and illuminated by ambient fluorescent ceiling lights. Mice were allowed free exploration of the arena for 10 min, during which time their movements were tracked (EthoVision, Noldus, Netherlands) and their ambulatory velocity, distance travelled, and rearing motions analyzed. Movement patterns were examined for the whole arena, as well as a pre-determined large outer square (20 cm^2^) and small inner square (center; 10 cm^2^). A 10% ethanol solution was used to clean the OF arena between each session.

### Elevated-plus Maze (EPM)

Mice were individually placed in a randomly selected closed arm of the EPM apparatus and permitted to explore the maze for 5 minutes (each of the four arms was 24.8 cm long and 7.7 cm wide; the two closed arms had opaque walls 21 cm high, and the other two arms had no walls). Each EPM session was camera recorded and scored for time spent and number of entries into the open arm, closed arm, and center, as well as for the frequency of stretching (keeping feet within closed arm or central area and extending head into to open arm) and head dipping (looking down over the side of the open arm). A 10% ethanol solution was used to clean the EPM between each session.

### Novelty Test

Mice were individually placed in the corner of a 30 cm^3^ opaque Plexiglas arena that contained a novel object (green candle holder, 18.0 cm). Mice were allowed free exploration of the arena for 5 minutes while a computer system (EthoVision, Noldus, Netherlands) tracked their movements and measured the latency to approach and the time spent in contact with the novel object.

### Immunohistochemistry

On day 14 of the experimental regimen, all mice (both the behaviourally tested and naïve cohort were included in order to ascertain whether the behavioural testing procedure itself might influence neurogenesis) were anaesthetized with 0.6 mL of pentobarbital (Ceva Sante Animale; lot 150A1) and perfused transcardially with saline followed by 4% paraformaldehyde (PFA) in 0.1 M Phosphate Buffer Saline (PBS) (Sigma-Aldrich). The brains were removed and stored at 4°C in the PFA mixture for 24 hours. This mixture was then replaced with a 20% sucrose solution (Sigma-Aldrich) and refreshed each day for 2 days, and then once a week for a month. The brains were then sliced to a thickness of 40 µm via the Cryotome FSE (Thermo Scientific). The slices were stained in the hippocampus for doublecortin (DCX). DCX was used as a marker to analyze the absolute number and dendritic growth of newly generated neurons in the adult dentate gyrus. On the first day of the DCX staining procedure, the slide-mounted brains were washed in a 0.01M PBS solution and then incubated in the primary goat anti-DCX antibody (1:200) at 4°C. Twenty four hours later the slides were washed again and then incubated for 2 hours in the secondary antibody, which was a donkey Alexa 488 anti-goat (1:100). Slides were then washed, air dried and cover-slipped. In order to quantify the number of DCX+ neurons within the dentate gyrus, bilateral counts from four hippocampal sections (bregma levels: -1.22, -1.58, -1.94 and -2.30) were pooled for each animal. All counts were done in a blinded fashion with the counter unaware of the treatments. BrdU staining, unfortunately, provided inconsistent outcomes, and hence the data were not assessed further.

### Role of mTOR in EPO induced behavioural changes

In a separate study, the mTOR pathway inhibitor, rapamycin, was used to determine whether EPO might induce antidepressant-like effects through this signaling pathway (as is the case with the novel antidepressant, ketamine). A separate cohort of 32 male CD-1 mice (10-12 weeks of age) received either saline or EPO (5000 U/kg ip.) treatment once every second day over a six day time period (i.e. three injections). These groups were subdivided so that half of the mice also received an injection of rapamycin (10 mg/kg ip, dissolved in saline with 5% DMSO), while the remaining animals received the vehicle (i.e. three injections) immediately prior to the EPO/saline injection. Three hours following the final injections, mice were tested in a FST paradigm identical to that described for the earlier experiment.

### Statistical Analysis

Prior to analyses, all data were checked for normality using the Shapiro-Wilk test with alpha = 0.05. Subsequently, it was found that none of the data violated the assumption of normality. Hence, behavioural data were analysed by a 2 factor (EPO injection × Social Stressor treatment) ANOVA, whereas immunohistochemical measures were assessed using a 3-factor ANOVA (EPO injection × Social Stressor × Prior Behavioural Testing) design. In the rapamycin study, a 2 (EPO vs vehicle) × 2 (rapamycin vs vehicle) ANOVA was used to assess FST performance data. Significant interactions were followed-up using Tukey post hoc comparisons. A StatView (SAS Institute, version 6.0) statistical software package was used for the computations.

## Results

### Experiment 1

#### Forced Swim Test

**Figure 1:**
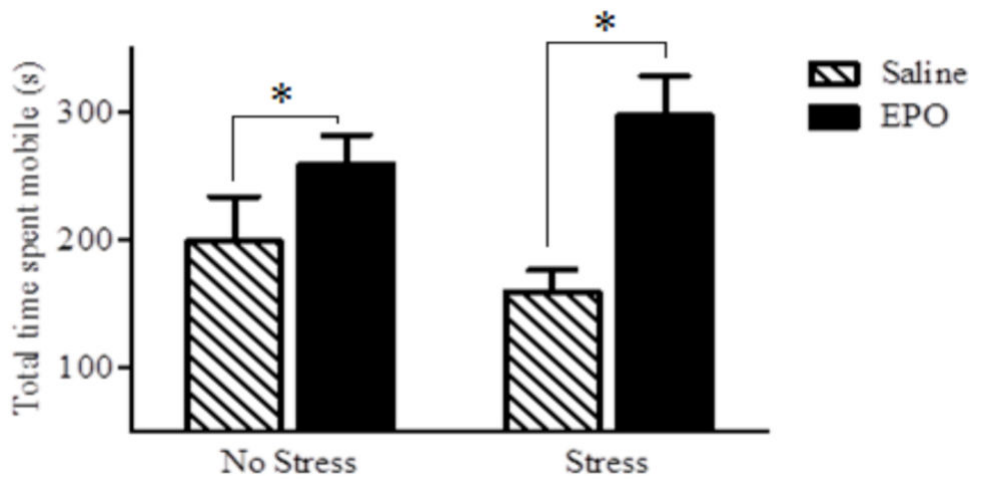
Time spent mobile, in seconds, in the forced swim test (FST). Data are expressed as mean ± SEM (*n* = 8/group). EPO (black bars) clearly reduced FST immobility relative to the saline treatment (hatched bars). This effect was apparent whether mice were exposed to the stressor regimen or not. *p < 0.001 relative to saline-treated controls.

There was no significant main effect of the stressor condition [*p* = 0.967], nor was there a significant interaction between the stressor condition and injection type on immobility time in the FST [*p* = 0.162]. However, the ANOVA revealed that EPO significantly reduced immobility in the forced swim test, irrespective of whether or not mice had been exposed to the stressor *F*(1,28) = 2.07, [*p* = 0.001] (Figure 1).

#### Open Field Test

**Figure 2:**
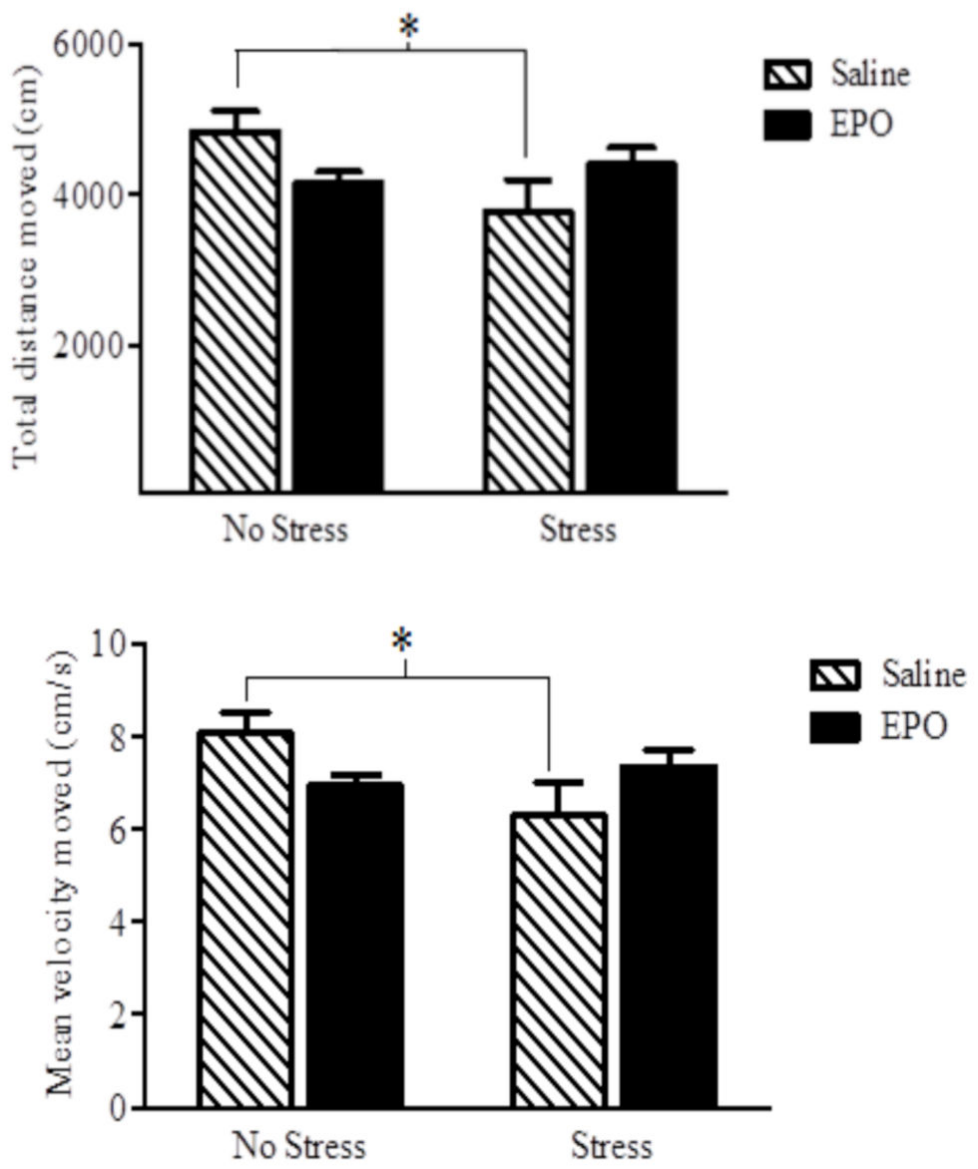
Total distance moved (top panel) and velocity of movement (bottom panel) in an open field (OF) arena. Stressor treatment significantly reduced both of these measures in the saline (hatched) but not the EPO (black bars) treated animals. Data are expressed as mean ± SEM (*n* = 8/group) *p < 0.05.

A significant EPO × stress interaction was apparent for both the total distance travelled in the open field, as well as the mean movement velocity *F*s(1,28) = 5.52 and 5.20, respectively, [*p* = 0.026]; See Figure 2. The follow-up comparisons confirmed that the stressor treatment reduced the total distance and velocity among the saline treated mice, whereas it had no effect in the EPO treated mice.

#### Elevated-plus Maze

**Figure 3:**
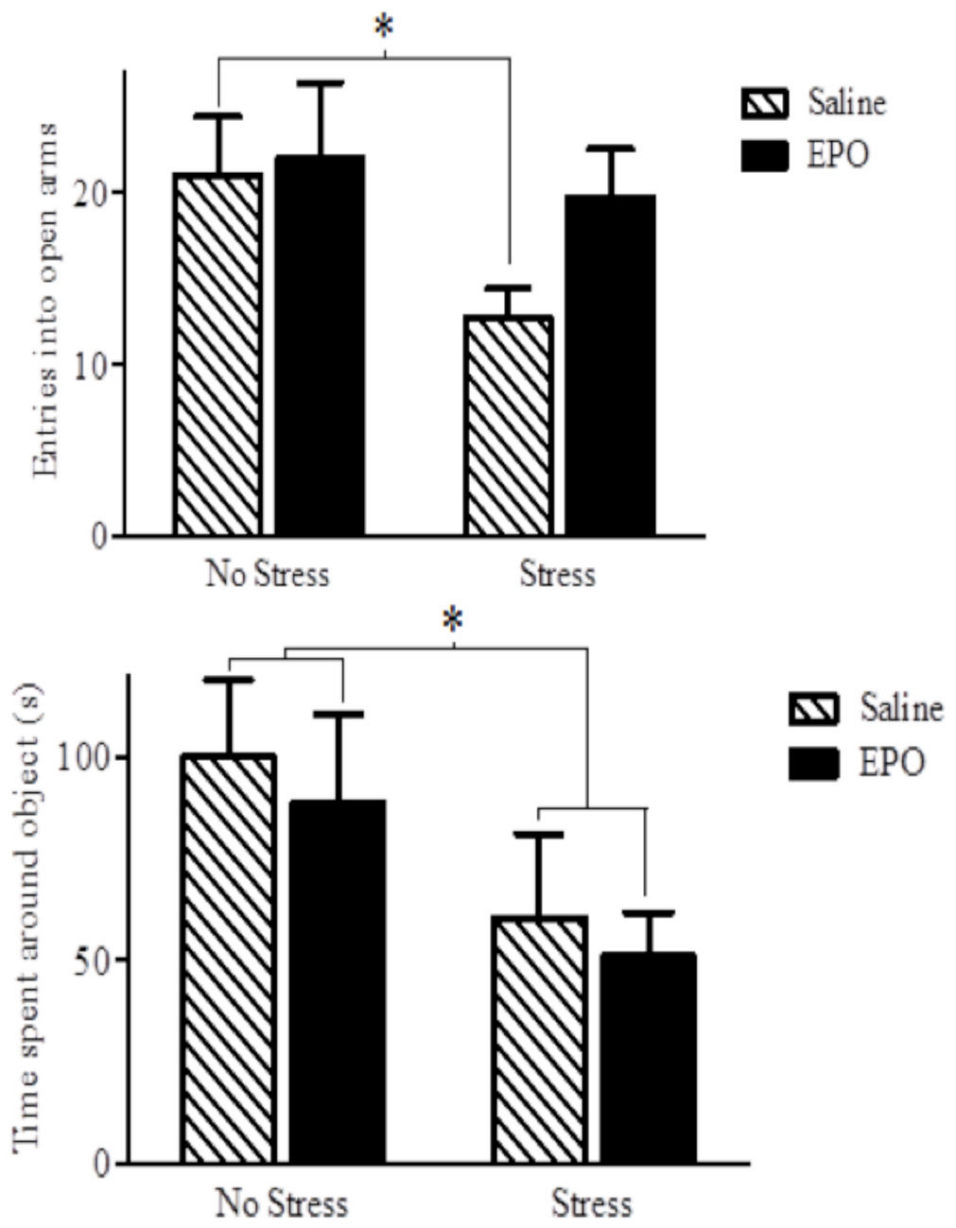
The number of entries into the open arm of an elevated plus maze (EPM) (top panel) and the time spent in contact with the novel object during a novelty task (bottom panel) The stressor treatment markedly reduced the number of EPM open arm entries in saline injected mice, but this effect was totally absent in mice that received the EPO treatment. The bottom panel depicts that a robust stressor-induced reduction of exploration of the novel object was apparent. However, EPO had no influence on this behavioural measure. Data are expressed as mean ± SEM (*n* = 8/group) *p < 0.05.

No significant differences were observed for either stress condition or injection group for rearing [*p* = 0.866, *p* = 0.380; respectively], stretch attends [*p* = 0.173, *p* = 0.080; respectively] and head dipping [*p* = 0.368, *p* = 0.388; respectively] in the EPM (data not shown). Similarly, for these EPM parameters no significant interaction was observed between EPO treatment and stressor [*p* = 0.199, *p* = 0.212, *p* = 0.348; respectively]. However, the number of entries into the open arms of the EPM was significantly reduced overall among the stressor-treated animals relative to the non-stressed controls *F*(1,14)= 4.51, [*p* = 0.020] (see Figure 3).

#### Novelty Test

The social stressor reduced the time spent in contact with the novel object located within the open field, *F*(1,29) = 4.51, [*p* = 0.005]. However, EPO administration had no effect on novel object exploration in either the stressed or non-stressed animals [*p* = 0.409] (Figure 3).

#### Immunohistochemistry

**Figure 4:**
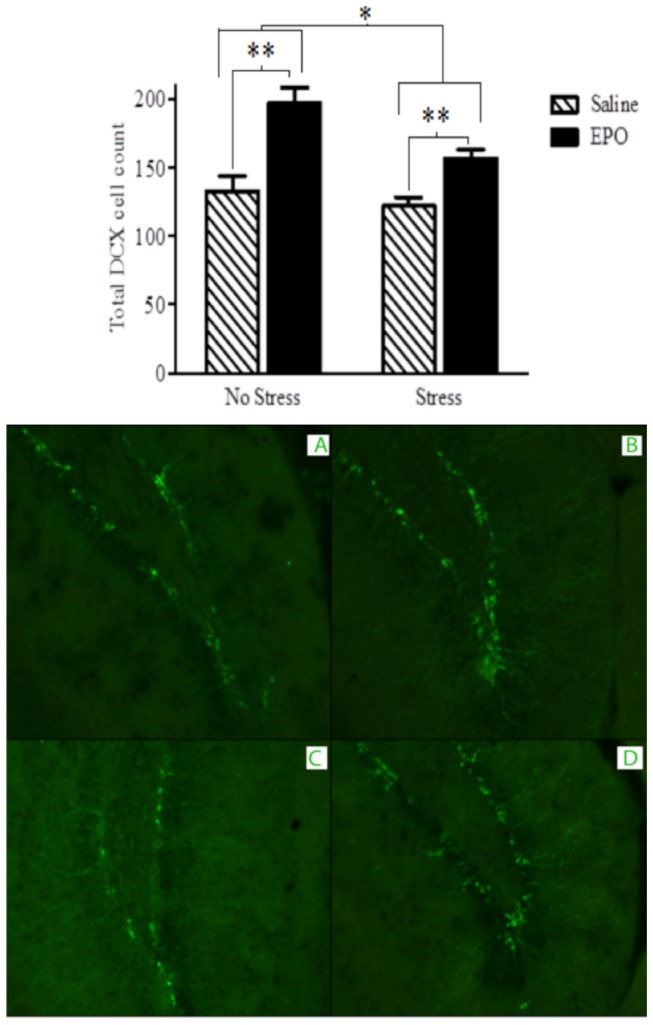
DCX immunoflourescent labelling of the dentate gyrus region of the hippocampus at 20X magnification. EPO treatment (black bars) increased DCX+ neuron counts relative to saline treatment (hatched bars). The bottom photomicrographs depict representative images from the treatment groups: A. Saline, B. EPO, C. Saline + Stress and D. EPO + Stress. Data are expressed as mean ± SEM (*n* = 8/group) *p < 0.01, **p < 0.001.

There were no main effects of [*p* = 0.413] or interactions involving behavioural testing and stress condition [*p* = 0.521] or behavioural testing and injection treatment [*p* = 0.200] on the number of DCX+ hippocampal neurons (Figure 4). There was also no significant three-way interaction between behavioural testing, stress condition and injection treatment [*p* = 0.904]. In the absence of any Stressor x EPO interactions [*p* = 0.118], there were significant main effects for both the stressor and EPO treatments. Specifically, the stressor induced a modest but significant reduction in DCX+ neurons *F*(1,24)= 28.62, [*p* = 0.009], whereas EPO treatment increased the number of DCX+ hippocampal neurons *F*(1,24) = 28.97, [p = 0.001], It is noteworthy that in the presence of the stressor, EPO increased DCX+ counts over and above that observed among saline treated animals (albeit to a lesser degree than that observed in the absence of the stressful challenge).

### Experiment 2

#### Rapamycin and forced swim test

**Figure 5:**
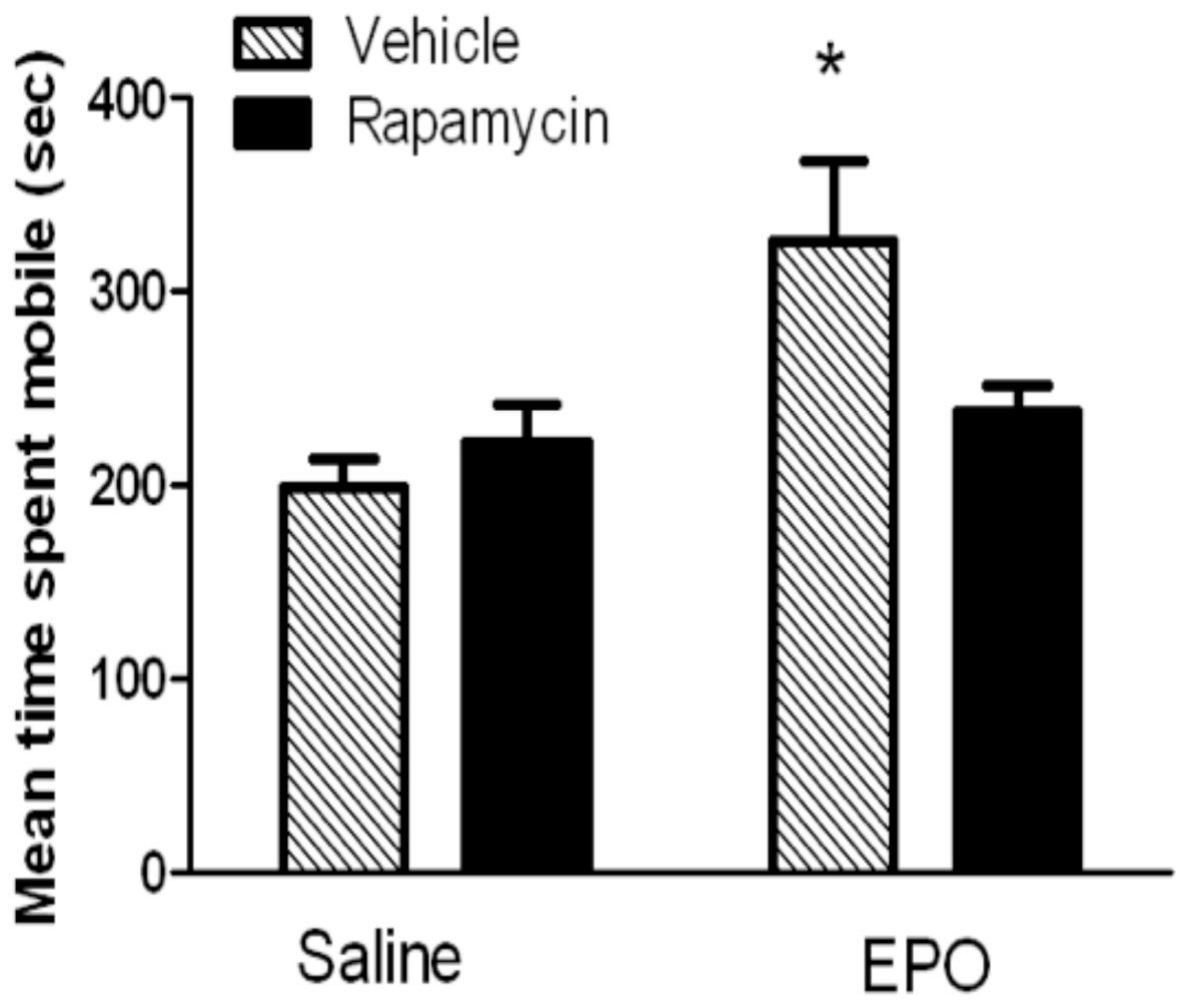
Rapamycin reversed the anti-depressant like effects of EPO in a forced swim test. As shown by the hatched bars, EPO treatment (5000 IU ip) reduced FST immobility time among the vehicle-injected mice. Rapamycin pre-treatment (black bars; 10 mg/kg ip), however, totally prevented the impact of EPO upon FST immobility. * *p* < 0.05.

A significant EPO × Rapamycin interaction was apparent with respect to time immobile in the FST *F*(1,26) = 5.19, [*p* = 0.03]. As shown in Figure 5, EPO treatment alone significantly reduced immobility in the FST (p = 0.01), whereas mice that also received the rapamycin injection did not differ from saline treated animals.

## Discussion

Our data showed that EPO can increase hippocampal neurogenesis and promote anti-depressant and anti-anxiety-like effects, and that mTOR might be an important mediator in at least some of these outcomes. Irrespective of the stressor treatment, EPO increased neurogenesis and reduction of immobility in the FST. Thus, rather than reversing the impact of the stressor, EPO alone appears to have potent neurogenic and anti-depressant-like (at least in this swim test) consequences. In addition, EPO did reverse the stressor-induced reduction of open field exploration and suppression of entries into the open arm of an elevated plus maze, suggesting that EPO can counteract anxiety-like effects induced by the stressor exposure. Finally, the fact that rapamyacin prevented the anti-depressant-like effects of EPO in the FST, indicates an importance of the mTOR pathway in underlying at least some of the effects of EPO.

As already mentioned, EPO had antidepressant-like effects as reflected by reduced immobility in the forced swim test (a common screening method of antidepressant activity). It has been suggested that the increased FST mobility might be due to the performance enhancing capabilities of EPO, given its ability to increase the presence of red blood cells [22]. However, in keeping with the findings of Girgenti et al. [24], we did not find a general EPO-related increase in mobility in the open field test. Thus, it is unlikely that the effects of EPO were attributable to potential motor enhancement. These antidepressant-like effects are consistent with earlier reports from Miskowiak and colleagues. Specifically, EPO was found to exert antidepressant-like effects in healthy and depressed individuals, in terms of behavioural and neural responses to emotional information, and such effects were apparent in the absence of any variations of reaction times [23,25,26,33–35].

Anxiety is frequently comorbid with depressive disorders [36–38] and SSRIs are often used to diminish anxiety [38]. Yet, there has been little data concerning the potential influence of EPO on behavioural indices of anxiety [39]. Nevertheless, Leconte et al. [40] recently reported that mild hypoxia, which is a known inducer of EPO, had anxiolytic-like effects in both the light/dark transition test and the EPM. However, in the Leconte et al. [40] report, EPO levels were not directly assessed, and an earlier study from this same group of investigators failed to find an anxiolytic-like effect of EPO [41]. In humans, a single high dose of EPO was found to reduce neural and cognitive responses to threat-relevant information 1 week following drug administration [25]. However, when participants were tested at an earlier time-point after EPO administration responses to threat were actually enhanced; this pattern of effects is similar to that seen with SSRIs [25].

In the current investigation EPO had no effect on the frequency of entries into the open arms of the EPM under the basal condition. However, EPO attenuated the reduction of open arm entries that was evident among mice receiving the social stressor. In effect, although the adaptive anxiety response to potentially threatening environmental stimuli or situations (e.g., the open arms of the EPM) may not be affected by EPO, the *excessive, abnormal* anxiety provoked by previous stressor experiences, which is ordinarily manifested in the EPM as a further reduction in exploration of the open arms, is effectively diminished by EPO. Curiously, analysis of movement patterns of mice in the open field test revealed that EPO attenuated the stressor-induced reduction in velocity and mean distance travelled in the open field arena as a whole. However, this pattern was not evident when movement patterns were assessed in the center of the arena. As the center of the arena is particularly apt in eliciting anxiety-related avoidance, it might be the case that the anxiolytic effects of EPO were limited. In fact, the results of the novelty test indicated that in this paradigm EPO did not attenuate the effects of the antecedent stressor. It has been fairly well established that several forms of anxiety should be considered in behavioural tests, such as the anxiety that occurs in response to specific stimuli vs that associated with contextual cues, as well as anxiety responses elicited by conditioned vs non-conditioned stressors as well naturalistic stressors [42]. Further studies are thus warranted to determine in greater detail whether or not EPO has anxiolytic properties.

Effective antidepressant treatments have been reported to increase hippocampal neurogenesis [43–45] and reverse the adverse effects of chronic stressors on neurogenesis [43,46]. It was similarly found in the present investigation that EPO increased hippocampal neurogenesis, as reflected by increased DCX staining. As this occurred irrespective of whether or not animals had been behaviourally tested, the increased neurogenesis cannot be attributable to learning effects associated with repeated testing. It was previously reported that chronic antidepressant treatment alone enhanced adult cell proliferation within the dorsal hippocampus [43–45], suggesting that these drugs have pro-mitotic effects independent of stressor conditions. The present results are consistent with this perspective, as EPO increased DCX staining regardless of whether or not mice had previously been exposed to a stressor. Finally, it should be mentioned that a few reports exist indicating that antidepressant treatments did not affect hippocampal neurogenesis and that the behavioral effects of antidepressants were independent of any influence on neurogenesis [47–49]. The reason for discrepancies between studies remains to be determined but might involve strain differences, as the Huang [48] and Holick [49] studies involved BALB/cJ mice, in contrast to the commonly used C57BL6 or CD1 (as in the present study) strains.

The increase in hippocampal neurogenesis may be a potential mechanism through which EPO exerts its antidepressant-like effects. Indeed, it has been suggested that the decreased hippocampal volume associated with depression [50–52], as well as the restoration of hippocampal volume with symptom remission [53], might be related to changes of neurogenesis [46,51,54–56]. As stressors suppress progenitor cells in the hippocampus [2,57–64], it follows that the enhancement of hippocampal neurogenesis by EPO could attenuate certain negative effects of stressors. In effect, the present findings contribute to the existing evidence indicating that the anti-depressant-like effects of EPO may be mediated, in part, by an augmentation of hippocampal neurogenesis.

The present findings suggest that the mTOR signaling pathway might be an important downstream process contributing to the forced swim alterations induced by EPO. Indeed, rapamycin completely inhibited the impact of EPO on FST performance, just as EPO was reported to attenuate the antidepressant effects of ketamine [65]. The present results are particularly interesting in light of the finding that mTOR underlies other positive effects upon neuroplasticity, including BDNF production, neurogenesis and synaptogenesis [32]. In addition to playing a neurogenic role in response to ketamine [32], the mTOR pathway was also found to be important for insulin or epileptogenic insult induced neurogenesis, as rapamyacin inhibited neurogenesis induced by these stimuli [66,67]. In the case of EPO, hippocampal EPO levels were elevated after effective antidepressant treatments [24] and EPO administration itself induced BDNF expression and adult hippocampal neurogenesis [28,29]. Thus, EPO might be eliciting its behavioural effects through mTOR- and- BDNF-associated enhancements of adult hippocamal neurogenesis and potentially other neuroplastic changes.

One limitation of this study is the fact that the FST is not a test for depressive-like behaviour per se, but rather a common screening method for antidepressant drugs. Yet, stressors and other treatments that promote depressive-like behaviours in other paradigms have also been reported to elicit increased immobility in the FST [68]. Another limitation would be that the effects of rapamyacin upon hippocampal neurogenesis were not determined. Thus, we cannot conclude as to whether the effects of inhibition of the mTOR pathway upon forced swim performance were related to hippocampal neurogenic processes. Finally, given that EPO did not significantly modulate the impact of the stressor (which itself had modest effects) on neurogenesis, it did not appear that EPO was directly targeting processes affected by the stressor.

Although the present investigation suggests that EPO could have beneficial anti-depressant or anti-anxiety like effects in the face of stressors, this cytokine might have clinically important effects for a range of other conditions in which neuroplasticity is disturbed. In this regard, EPO protected hippocampal neurons from stressor-induced apoptosis [69–71] and rescued hippocampal CA1 neurons from ischemic damage; EPO was also reported to attenuate performance disturbances in the Morris water maze [72,73]. Chronic EPO treatment might even have cognitive enhancing effects, as indicated by improvements in spatial performance in the Morris water maze test [22] and the improved executive functioning, coding and working memory, and psychomotor speed observed in EPO treated multiple sclerosis and schizophrenic patients [74]. Translating findings from animal models of depression into human clinical trials can be challenging and is not always successful. In the case of EPO, caution would need to be exercised with respect to dose, particularly in light of the potential for thrombosis or other hematopoietic complications. However, EPO is currently being used successfully to treat anemia without apparent toxicity as an obstacle for its use. The present findings contribute to the emerging evidence that EPO may be a new candidate treatment for affective disorder, or at least be useful as an adjunctive agent together with existing treatments.

## Supporting Information

Table S1 Experimental design and timeline of treatments and behavioral testing. (DOC)
