# Supplementary figures and images for: Antidepressant-Like Effects of Erythropoietin: A Focus on Behavioural and Hippocampal Processes
Source: PLoS One. 2013 Sep 3;8(9):e72813. doi: 10.1371/journal.pone.0072813 (PMC3760922; doi:10.1371/journal.pone.0072813)

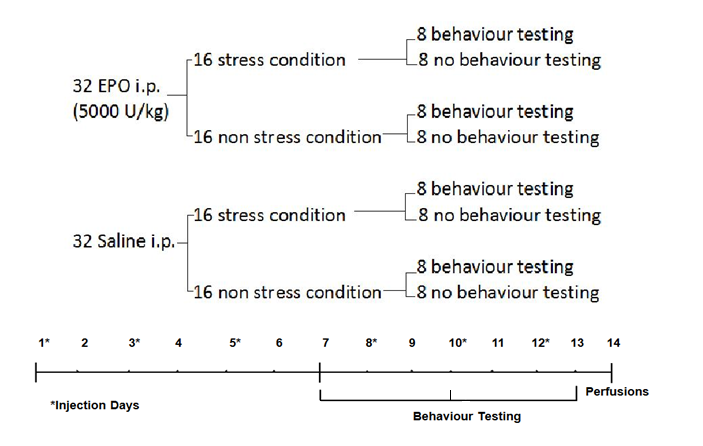

Supplement: Table S1 — (DOC) [file pone.0072813.s001.doc]
